# Restricted cafeteria feeding and treadmill exercise improved body composition, metabolic profile and exploratory behavior in obese male rats

**DOI:** 10.1038/s41598-022-23464-7

**Published:** 2022-11-15

**Authors:** Adam Alvarez-Monell, Alex Subias-Gusils, Roger Mariné-Casadó, Xavier Belda, Humberto Gagliano, Oscar J. Pozo, Noemí Boqué, Antoni Caimari, Antonio Armario, Montserrat Solanas, Rosa M. Escorihuela

**Affiliations:** 1 Medical Physiology Unit, Department of Cell Biology, Physiology and Immunology, Faculty of Medicine, Universitat Autònoma de Barcelona, 08193 Bellaterra, Spain; 2 Institut de Neurociències, Universitat Autònoma de Barcelona, 08193 Bellaterra, Spain; 3 Unitat de Psicologia Mèdica, Departament de Psiquiatria i Medicina Legal, Universitat Autònoma de Barcelona, 08193 Bellaterra, Spain; 4 Eurecat, Centre Tecnològic de Catalunya, Biotechnology Area and Technological Unit of Nutrition and Health, 43204 Reus, Spain; 5 Animal Physiology Unit, Department of Cell Biology, Physiology and Immunology, Faculty of Biosciences, Universitat Autònoma de Barcelona, 08193 Bellaterra, Spain; 6 Gastroesophageal Carcinogenesis Group, IMIM (Hospital del Mar Medical Research Institute), Carrer Doctor Aiguader 88, 08003 Barcelona, Spain

**Keywords:** Neuroscience, Physiology

## Abstract

The aim of this study was to evaluate, in male Long-Evans rats, whether a restricted-cafeteria diet (CAFR), based on a 30% calorie restriction vs continuous ad libitum cafeteria (CAF) fed animals, administered alone or in combination with moderate treadmill exercise (12 m/min, 35 min, 5 days/week for 8 weeks), was able to ameliorate obesity and the associated risk factors induced by CAF feeding for 18 weeks and to examine the changes in circadian locomotor activity, hypothalamic–pituitary–adrenal (HPA) axis functionality, and stress response elicited by this dietary pattern. In addition to the expected increase in body weight and adiposity, and the development of metabolic dysregulations compatible with Metabolic Syndrome, CAF intake resulted in a sedentary profile assessed by the home-cage activity test, reduced baseline HPA axis activity through decreased corticosterone levels, and boosted exploratory behavior. Both CAFR alone and in combination with exercise reduced abdominal adiposity and hypercholesterolemia compared to CAF. Exercise increased baseline locomotor activity in the home-cage in all dietary groups, boosted exploratory behavior in STD and CAF, partially decreased anxiety-like behavior in CAF and CAFR, but did not affect HPA axis-related parameters.

## Introduction

The prevalence of obesity and overweight is increasing and poses a major health burden worldwide^1^. Obesity has several associated metabolic and hormonal alterations, which lead to Metabolic Syndrome (MetS) and increase cardiovascular and cancer risks among others^2,3^. Several behavioral alterations have also been described, including circadian dysregulation^4,5^, altered cognitive function^6,7^ and anxiety^8,9^, both in rodents and humans.

Several experimental models have been used to study obesity^10^. Cafeteria (CAF) diet, in which animals are fed several high-fat and high-sugar components of human diets, is one of the most robust models of diet-induced obesity (DIO)^11,12^. It is a model with face validity for human obesity^13^, inducing hedonic eating due to the high palatability of its ingredients, and leading to altered eating behavior such as snacking and hyperphagia^11,12,14,15^. Moreover, CAF diet generates more severe MetS symptoms than other experimental diets^16,17^.

CAF diet simulates the intake of comfort food in humans, which has been shown to have an anxiolytic effect and to decrease the glucocorticoid response^18^. In rats, seven days of palatable food intake reduced plasma adrenocorticotropic hormone (ACTH) and corticosterone responses to restraint stress^19^. Studies have shown that, rather than the macronutrient composition, it is the choice, liking, and palatability of the food that induces these effects, both in rodents^20^ and in humans^21^.

Clinically, obesity is treated through dietary and exercise interventions, aimed at reducing energy intake and increasing energy expenditure, anti-obesity medications, and bariatric surgery in severe cases^22^. The widely used dietary interventions consist of changing diet quantity and quality to decrease adiposity and MetS markers^23,24^. However, these interventions tend to have low adherence^25^ and can generate stress and anxiety due to lower diet satisfaction^8^.

We have characterized a calorie-restricted CAF (CAFR) diet based on the same palatable foods of CAF but with a 30% calorie restriction relative to the energy intake of CAF animals. A 30% was chosen because 15–30% restriction over the usual diet is the recommended for dieting in human obesity management^26^. It has been proposed as a realistic and precise approach to evaluate the anti-obesity effect of a caloric restriction-based intervention combined with other therapeutic strategies. This diet decreases energy intake and ameliorates the biometric, metabolic, and hormonal imbalances associated with MetS^27^. We have also shown that CAFR diet, especially when supplemented with the polyphenolic compound oleuropein, has additional beneficial effects on sweet taste function, which is altered in obesity^28^. This diet models obese and overweight individuals with partial success and eating relatively often the not recommended cafeteria products while dieting. The little portions of those cafeteria items would increase and maintain adherence.

A corrective intervention such as physical exercise generates an overall healthier profile by improving cardiovascular function and decreasing the risk of developing type 2 diabetes among other benefits^29–31^. Moderate combined aerobic and resistance exercise programs can modestly contribute to weight loss and metabolic health, which are mainly induced by low-calorie diets^32^, and have been shown to reduce depression and stress scores in humans^33^. The Norwegian HUNT cohort study involved a healthy cohort of 33,908 adults which were followed up for 11 years and linked regular leisure-time physical activity (LTPA; walking, jogging, swimming) to a reduced incidence of depression^34^.

In rats, the effects of treadmill exercise on body weight are inconsistent, with publications reporting a mild decrease^35^ or no effects^36,37^. However, regardless of body weight changes, an improvement in obesity-related metabolic dysfunctions (muscle triacylglycerides content or renal function) has been shown^35,37^. More specifically and pertaining to the task at hand, treadmill exercise in rats reverted impairments in stress-coping induced by CAF feeding and decreased anxiety-like behaviour^36^.

Here we aimed at characterizing the effects of CAF and CAFR feeding as well as an exercise intervention upon biometric and metabolic parameters, circadian locomotor activity, exploratory and anxiety-like behavior, and HPA axis function in obese male Long-Evans rats. We hypothesized that CAFR would partially revert the effects of *ad libitum* (*a.l.*) CAF induced behavioral and hormonal changes in the HPA axis, and that treadmill exercise should further palliate the obese phenotype in CAFR fed animals to a greater degree than in CAF fed animals. We used an experimental design in which CAF diet was administered for 10 weeks from weaning onwards to induce obesity compared to standard chow, and then dietary and exercise interventions were applied for 8 weeks, after which behavioral testing was performed.

## Methods

### Animals

We used 60 male Long-Evans rats (Janvier, France) 23–25 days-old upon arrival. Animals were housed in pairs in standard Plexiglas cages, maintained under a 12:12 h light–dark cycle (lights on at 8 h) in standard conditions (temperature: 21 ± 1 °C; humidity 50 ± 10%) and allowed 1 week of habituation to the animal room. After the habituation, they were randomly assigned to two groups balancing mean body weight (BW): Standard (STD), n = 20, BW = 79.58 ± 2.03 g; and CAF, n = 40, BW = 79.60 ± 1.36 g. From week 10 onwards, animals were housed individually to measure diet consumption. The experimental protocol was approved by the Generalitat de Catalunya (DAAM 9978), following the ‘Principles of laboratory animal care’ and was performed in accordance with the European Communities Council Directive (2010/63/EU). The present study is reported in accordance with ARRIVE guidelines.

### Experimental design

**Figure 1:**
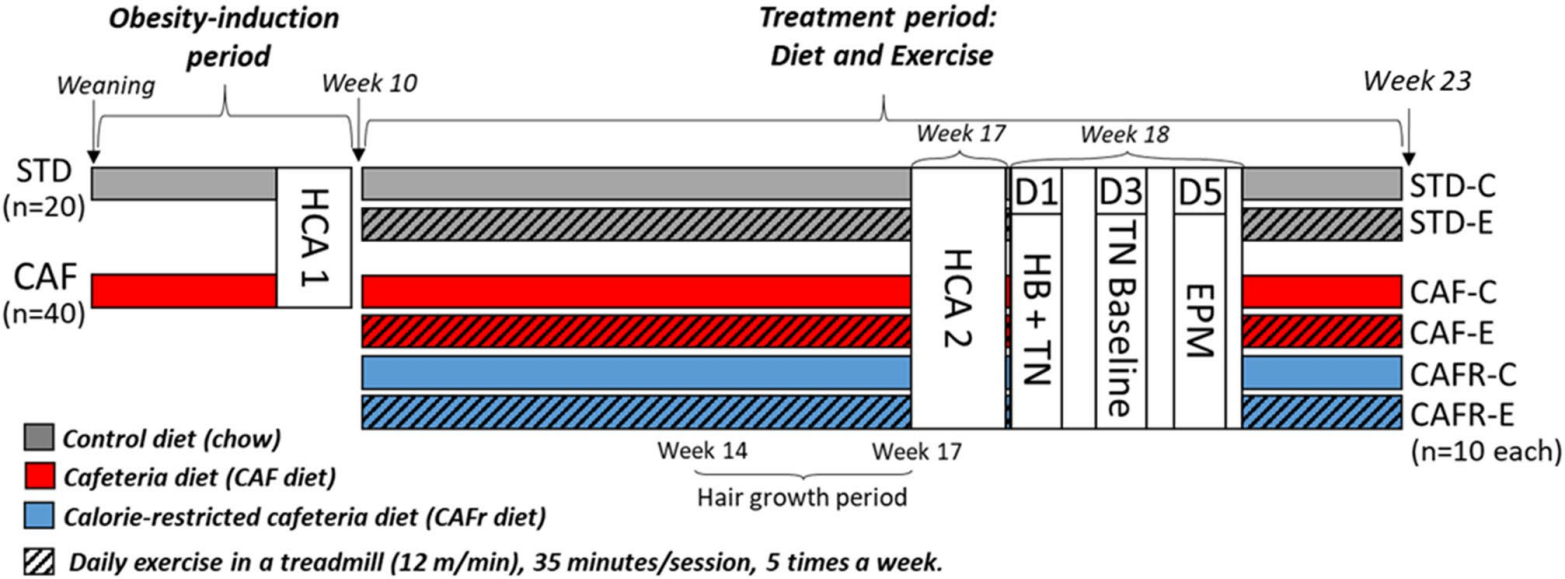
Experimental design showing groups, diets, and behavioral tests. Hair growth period refers to the period in which hair was allowed to regrow to be collected for the hair corticosterone study, see “Hair corticosterone” for details. *HCA* Home-Cage Activity; *HB* Hole Board; *EPM* Elevated Plus Maze; *TN* Tail nick.

We used a 3 × 2 factorial design with Diet (STD, CAF, and CAFR) and Exercise (Control -C- and Exercise -E-) as factors (Fig. 1). Once obesity had been induced by the administration of CAF diet for 10 weeks, animals were randomly allocated to the following groups, balancing mean BW: STD-C, n = 10, BW = 373.2 ± 6.1 g; STD-E, n = 10, BW = 374.7 ± 5.2 g; CAF-C, n = 10, BW = 413.9 ± 10.0 g; CAF-E, n = 10, BW = 412.0 ± 8.2 g; CAFR-C, n = 10, BW = 413.4 ± 10.1 g; and CAFR-E, n = 10, BW = 411 ± 6.3 g. Interventions were maintained throughout the experiment. Animals were weighed weekly, and nose-to-anus length (NAL) and abdominal circumference (AC) were measured every two weeks. Lee Index [((BW(g)^1/3)/(NAL(cm)·10))·10000] was also calculated biweekly. Food intake was calculated every week as the difference between food provided and the remainder the day after. At weeks 9 and 17 we performed the home cage activity (HCA) test and at week 18 the hole board (HB) and the elevated plus maze (EPM) tests. At week 23 animals were sacrificed by decapitation after an 8-h fast. Fasting started in the morning at 7 h, leaving 20 min between animals, and sacrifices were carried out between 15 and 18 h. The following tissues were collected and weighed: adrenal glands, thymus, white adipose tissue -WAT- depots (inguinal, retroperitoneal, epididymal and mesenteric), and soleus and gastrocnemius muscles. Blood was collected and serum was obtained by centrifugation at 2000 g for 15 min and stored at − 80 °C until further analysis. Between weeks 19 and 22 animals performed a set of behavioral tests unrelated to the present study being prepared for publication in an independent paper.

### Diets

During the obesity-induction period of the experiment, the STD group was fed standard chow *a.l.* whereas the CAF group was fed CAF diet a.l., and all animals had access to tap water *a.l.*. The amount of CAF items provided was progressively raised during the first 8 weeks to match the growth of the animals (Table S1). From week 10 onwards, the CAF diet consisted of (average quantities per rat/day): bacon (8 g), biscuits + pâté (5 g), biscuits + cheese (5 g), muffins (7 g), carrots (8 g), jellied sugared milk (a mixture of milk, sugar, and neutral gelatine with a solid consistency) (45 g), and standard chow (25 g). The amount of food and energy administered daily were 103 g and 246 kcal respectively, and the caloric distribution was: 10.5% protein, 39% fat and 50.5% carbohydrates. The caloric content of the standard chow (Teklad Global 14% protein rodent diet 2014, Envigo, US) was 290 kcal per 100 g, and its caloric distribution was: 20% protein, 13% fat and 67% carbohydrates.

The CAFR diet was based on the same items as the CAF diet, but the amount of each item was readjusted weekly to match a 30% calorie-restriction relative to the mean energy intake of the CAF group. The quantities of CAF items provided were (averaged quantity per rat/day): bacon (3.4 g), biscuits + pâté (3.3 g), muffins (1.8 g), carrots (7.6 g), jellied sugared milk (12 g) and standard chow (13 g). The average amounts of food and energy administered per day were 41 g and 97 kcal respectively, and the caloric distribution was: 12% protein, 38% fat and 50% carbohydrates. All food was renewed daily.

### Treadmill training

Training was performed in a treadmill (Columbus instruments, Columbus, OH, USA) placed in a separate room with mild lighting. The apparatus consisted of 3 separated parallel runways (45 × 11 × 12 cm), topped by Plexiglas. Two identical apparatuses were used for the training with one animal from each diet group. Non-exercised animals were placed in the treadmills at an intensity of 0 m/min. This exercise paradigm was chosen over others in order to ensure all animals followed the same training procedure in terms of frequency, session duration and number, and intensity.

Training began at week 10, with daily training sessions 5 days/week (from Sunday to Friday). No training was performed during the behavioral testing days. The intensity was raised progressively from sessions 1 to 12 (Table S2). From session 13 onwards, all sessions started at an intensity of 6 m/min which was raised 1 m/min every minute until reaching an intensity of 12 m/min. From that point on, the intensity was maintained until minute 30, during which the animals ran approximately 341 m, then the intensity was lowered to 8 m/min. Sessions ended at minute 35, at an approximate total distance of 381 m. This intensity was chosen since animals can perform this exercise without needing aversive stimulation to run (i.e. electric shock)^36,38^; and higher intensities can be considered an stressor on the animals^39^. Eight weeks were chosen as the duration of the intervention since similar durations have provided positive results previously^36^.

### Serum analyses

Enzymatic colorimetric kits were used for the determination of serum total cholesterol, triglycerides, and glucose (QCA, Barcelona, Spain), HDL-cholesterol and LDL-cholesterol (Bioassay systems, CA, USA) and non-esterified free fatty acids (NEFAs) (WAKO, Neuss, Germany). Serum insulin and leptin levels were measured using a mouse/rat insulin ELISA kit (Millipore, Barcelona, Spain) and a rat leptin ELISA kit (Millipore). The homeostasis model assessment-estimated insulin resistance (HOMA-IR) was calculated following the formula^40^: HOMA-IR = Glucose(mM) X Insulin(mU/L)/22.5.

### Behavioral testing

#### Home cage activity (HCA)

Two identical sound-proof HCA apparatuses were used to determine basal circadian locomotor activity at weeks 9 and 17 (Fig. 1). Each apparatus contained two identical home-cages (40 × 23 × 18 cm) made from transparent Plexiglas (Med Associates Inc., USA). Animals were tested from 15 to 10 h of the following day, with normal light cycles. An infrared camera 43 cm above the top of the cages recorded the session and the computer recorded the distance travelled (cm) in 1 min bins that were collapsed to obtain the distance travelled each hour. Data were analysed into two periods, one for the lights on period (ON, from 16 to 19 h and from 8 to 10 h) and one for the lights OFF period (OFF, from 20 to 07 h). Data for 15 h was not considered due to high distances recorded because of the novel environment exposure in the testing home-cage. The corresponding diets and water were administered normally at the start of the test. The test was carried out in a different room.

#### Exploratory activity and anxiety-like behavior

Behavioral testing was performed at week 18 between 9 and 15 h. The HB test was administered on day 1 and the EPM on day 5 (Fig. 1). The apparatuses were cleaned with 70% ethanol between animals and dried with a paper towel.

Two HB apparatuses were used (66 × 66 × 47 cm) made of white wood and each containing four equidistant holes (3,7 cm diameter, 18 cm depth) on the floor. The animals were placed in the apparatus facing a corner and recorded for the following 15 min, measuring the distance travelled, the number of rearings, and the exploratory activity, by means of the head dips and time spent head-dipping (HD). A HD was defined as “introducing the head into the hole below eye level”.

The EPM apparatus consisted of four arms (50 × 10 cm) made of white wood, extending from a square centre (10 × 10 cm), and positioned at 90° angles from each other. Two opposing arms had wooden walls (40 cm high), whereas the other two arms were open, with a 0.5 cm ridge for additional grip. The whole apparatus was elevated 50 cm above the floor. At the start of each session the animal was placed in the centre of the maze facing an enclosed arm and for 5 min we scored: the number of enclosed and open arm entries, the time spent, and distance travelled into each arm, and number of entries in the central square. A zone entry/exit was defined as “placing the four paws into/out of a given arm”.

All tests were recorded with a video camera and the videos were analysed with a video tracking software (ANY-Maze, SD Instruments, US) in a semi-automatic way by a trained researcher. For the HB, the experimenter manually recorded the number of rearings and exploratory behaviors, while the software obtained the distance travelled. For the EPM, the experimenter manually recorded arm entries and the software recorded the time spent and the distance travelled into each arm.

### HPA axis analyses

#### Plasma ACTH and corticosterone

Fifteen minutes of HB exposure allowed us to evaluate the HPA response to a mild stressor (novel environment). This time was chosen because it is the minimum time needed for plasma corticosterone to reflect initial ACTH release^41^. Sampling for basal hormones was performed under resting conditions in the morning (9–10 h, AM sample) and evening (20–21 h, PM sample) (day 3 week 18, Fig. 1). Blood samples were taken by tail nick, performed by gently wrapping the animals with cloth, making a 2 mm incision at the end of the tail vein and massaging the tail while collecting 300µL of blood into ice-cold EDTA capillary tubes. Animals that performed the HB simultaneously were sampled together for all samples. All samplings were performed in a separate room. Simultaneous sampling of two animals was performed by two trained experimenters.

#### Hair corticosterone

Sampling was performed at week 17 to assess mid- to long-term activity of the HPA axis. Hair was removed from the posterior third of the animals’ dorsal side at week 14, allowed to regrow for 3 weeks, and after this hair growth period, new hair was collected from the shaved area and stored at 4 °C. Sampling was performed in a separate room. Hair samples were homogenized and 40 mg of hair were weighed and placed into 5 mL glass tubes. Samples were washed 3 times with 4 mL of undiluted 2-propanol (Sigma, Spain) using an orbital tube mixer (360° rotation at 20 rpm, Minilab Roller, Sigma) for 3 min, followed by centrifugation (2000 g, 10 min) and removal of the supernatant. Residual 2-propanol was evaporated in a dry bath at 50 °C overnight. Then, 1.6 mL of HPLC-grade methanol (Scharlau, Spain) was added and mixed at room temperature by rotation (20 rpm) overnight. After, the methanol was collected into 2 mL Eppendorf safe-lock tubes and dried in a vacuum centrifuge for 2.5 h until the methanol evaporated. We performed three washes with methanol. Dried samples were stored at − 20 °C and resuspended with 1 mL 0.1 M phosphate buffer.

### Radioimmunoanalyses

Plasma levels of corticosterone and ACTH were assayed by double anti-body radioimmunoassay (RIA). In brief, corticosterone RIA used 125I-corticosterone-carboximethyloxime-tyrosine-methyl ester (ICN-Biolink 2000, Spain), synthetic corticosterone (Sigma) as the standard, and an antibody raised in rabbits against corticosterone-carboximethyloxime-BSA, provided by Dr. G. Makara (Inst. Exp. Med., Hungary). The characteristics of the antibody and the RIA procedure have been previously described^42^. The RIA protocol recommended by Dr. Makara (plasma corticosteroid-binding globulin was inactivated by low pH) was followed. ACTH RIA used 125I-ACTH (PerkinElmer Life Science, Boston, USA), synthetic ACTH 1–39 (Sigma) as the standard, and an antibody raised in rabbits against rat ACTH (rb7) provided by Dr. W.C. Engeland (Department of Surgery, University of Minnesota, Minneapolis, USA). The characteristics of the antibody have been previously described^43^. All directly compared samples were processed in the same assay.

### Liquid chromatography tandem mass spectrometry (LC–MS/MS)

Hair corticosterone levels were determined based on a previously described methodology^44^. Briefly, 1.5 mL of saturated NaCl and 6 mL of ethyl acetate were added to hair extracts. After performing a liquid–liquid extraction, the organic layer was evaporated and reconstituted with 150 µL of water:methanol 50:50. Finally, 10 µL of the reconstituted extracts were injected into the LC–MS/MS System. Corticosterone was quantified by external calibration using d6-corticosterone as an internal standard.

### Statistics

Statistical analysis was performed using the “Statistical package for Social Sciences” (SPSS, version 22, IBM, US). Data were checked for homoscedasticity and normality with Levene’s and Kolmogorov–Smirnov tests, respectively. Data at week 10 (BW, Lee Index, AC, mean energy intake and HCA data) were analysed with the Mann–Whitney U test due to different group sizes. Data collected at one time point (BW, Lee Index, AC, energy intake, adrenal gland and thymus weight, hormonal response to the HB, HB and EPM and hair corticosterone) were analysed with 2 two-way ANOVAs, the first one comparing the groups STD and CAF [diet (STD, CAF) x exercise (C, E)], and the second one comparing the groups CAF and CAFR [diet (CAF, CAFR) x exercise (C, E)]. This approach allowed better determination of the effects of CAF feeding and exercise compared with STD and control animals, and separation of the effects of CAFR feeding and exercise compared with the obese CAF fed animals. In case significant diet effects appeared on the 2nd two-way ANOVA revealing that CAFR feeding might have beneficial effects on obese cafeteria fed animals, an additional two-way ANOVA comparing STD and CAFR diets was performed. This final analysis allowed us to determine whether CAFR feeding had returned obese animals to the STD conditions or there were still differences between them. Finally, data comprising several time points (HCA, basal hormones) were also analysed with 2 two-way repeated measures ANOVAs, with time as the within-subject factor and diet and exercise as the between-subjects factors. Both within-subject and between-subject factor analyses allowed detection of differences over the time points and overall, respectively.

## Results

### CAFR Feeding and exercise palliated the effects of CAF feeding on biometric parameters

**Figure 2:**
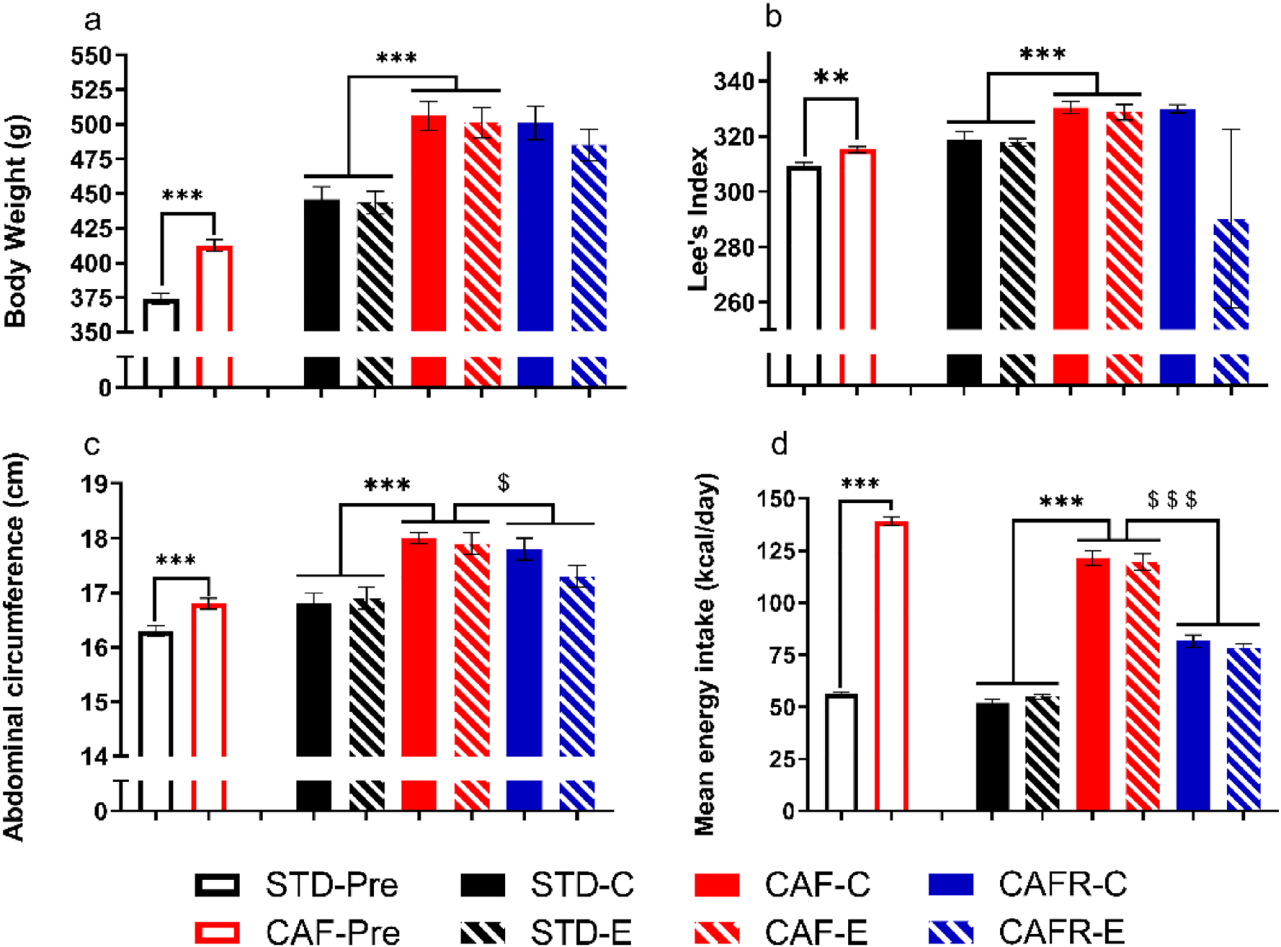
Effects of dietary interventions and exercise on biometric parameters and mean energy intake. (**a**) Body weight (g). (**b**) Lee Index. (**c**) Abdominal circumference (cm). (**d**) Mean energy intake (Kcal/day). Empty bars are data at the end of the obesity-induction period before the start of interventions (week 10); and solid and dashed bars are data from the time of behavioral testing (week 18). Values are mean ± SEM. **p < 0.01, ***p < 0.001 versus STD; $*p* < 0.05, $$$*p* < 0.001 versus CAF.

We first characterized the effects of CAF feeding upon several biometric and energy intake parameters at week 10, immediately before the interventions, and week18, immediately before behavioural testing (Fig. 2).

At week 10, CAF fed animals showed higher BW, Lee Index and AC than STD fed animals [BW: U = 91; *p* < 0.001; Lee Index: U = 203, *p* = 0.003; AC: U = 202; *p* = 0.003]; and displayed a 2.46-fold increase in mean daily energy intake [U = 55; *p* < 0.001]. At week 18, CAF feeding increased BW, Lee Index, AC and the average energy intake compared to STD feeding [diet: BW: F(1,39) = 36.981, *p* < 0.001; Lee Index: F(1,39) = 23.634, *p* < 0.001; AC: F(1,39) = 35.610, *p* < 0.001; average energy intake: F(1,39) = 599.540, *p* < 0.001]. Neither exercise nor the diet*exercise interaction exerted any effect upon these variables.

On the other hand, CAFR significantly decreased AC and mean energy intake [diet: AC: F(1,37) = 4.980, *p* = 0.033; energy intake: F(1,37) = 599.54, *p* < 0.001] compared to CAF. No effect on BW or Lee Index was found. No exercise or diet*exercise effects were found.

### CAFR feeding and exercise improved body composition and serum obesity-related biomarkers in obese animals

As shown in Table 1, CAF feeding increased all WAT depots (inguinal, retroperitoneal, mesenteric and epididymal), both in absolute (g) and relative weight (g/100 g of body weight) compared to STD. On the other hand, CAF feeding decreased relative weight of the soleus and gastrocnemius skeletal muscles but did not affect their absolute weights. CAF feeding also decreased the relative weight of the adrenal gland but did not affect the thymus weight. Regarding exercise in STD and CAF fed animals, it decreased relative retroperitoneal adiposity, and more notably the relative total abdominal WAT (sum of retroperitoneal, epididymal and mesenteric fat depots).

**Table 1:** Effects of dietary interventions and exercise on the absolute (g) and relative weight (g/100 g) of white adipose depots, skeletal muscle, adrenal gland and thymus at the end of the experiment.

	STD-C	STD-E	CAF-C	CAF-E	CAFR-C	CAFR-E	1st ANOVA (STD vs. CAF)	2nd ANOVA (CAF vs. CAFR)
**WAT absolute weight (g)**								
Inguinal	21.78 ± 1.46	19.68 ± 1.72	36.26 ± 1.84	38.33 ± 3.01	35.60 ± 1.56	29.64 ± 1.91	D***	D*
Retroperitoneal	10.90 ± 0.57	8.92 ± 0.65	20.37 ± 1.12	19.23 ± 0.93	17.32 ± 0.70	16.46 ± 0.99	D***	D**
Mesenteric	8.18 ± 0.47	7.33 ± 0.67	12.32 ± 0.53	11.47 ± 0.60	10.56 ± 0.39	10.58 ± 0.48	D***	D*
Epididymal	7.42 ± 0.24	6.68 ± 0.33	12.14 ± 0.65	12.41 ± 0.50	11.32 ± 0.35	11.00 ± 0.36	D***	D*
Abdominal	26.50 ± 1.16	22.93 ± 1.56	44.84 ± 1.96	43.12 ± 1.87	39.21 ± 1.27	38.05 ± 1.67	D***	D**
Total	48.28 ± 2.54	42.61 ± 3.12	81.11 ± 3.50	81.45 ± 4.83	74.81 ± 2.57	67.70 ± 2.86	D***	D**
**Muscle absolute weight (g)**								
Soleus	0.18 ± 0.01	0.17 ± 0.01	0.18 ± 0.01	0.17 ± 0.01	0.17 ± 0.01	0.17 ± 0.01	–	–
Gastrocnemius	2.30 ± 0.04	2.29 ± 0.02	2.36 ± 0.05	2.31 ± 0.07	2.23 ± 0.05	2.18 ± 0.05	–	D*
**Adrenal gland absolute weight (mg)**	39.50 ± 2.64	40.40 ± 2.15	39.89 ± 2.37	41.33 ± 3.23	40.44 ± 1.04	38.56 ± 2.09	–	–
**Thymus absolute weight (mg)**	135 ± 4	124 ± 9	151 ± 12	147 ± 12	150 ± 9	128 ± 7	–	–
**WAT relative weight (g/100 g)**								
Inguinal	4.57 ± 0.25	4.15 ± 0.29	6.51 ± 0.27	6.84 ± 0.40	6.63 ± 0.29	5.64 ± 0.32	D***	–
Retroperitoneal	2.29 ± 0.09	1.88 ± 0.11	3.66 ± 0.17	3.45 ± 0.11	3.23 ± 0.14	3.12 ± 0.15	D***, E*	D*
Mesenteric	1.72 ± 0.08	1.54 ± 0.11	2.21 ± 0.06	2.05 ± 0.07	1.96 ± 0.06	2.01 ± 0.05	D***	D*
Epididymal	1.56 ± 0.03	1.41 ± 0.06	2.18 ± 0.09	2.23 ± 0.05	2.11 ± 0.06	2.09 ± 0.06	D***	–
Abdominal	5.58 ± 0.11	4.85 ± 0.26	8.05 ± 0.25	7.73 ± 0.19	7.31 ± 0.24	7.23 ± 0.22	D***, E*	D*
Total	10.15 ± 0.41	9.01 ± 0.52	14.57 ± 0.45	14.58 ± 0.58	13.94 ± 0.48	12.87 ± 0.39	D***	D*
**Muscle relative weight (g/100 g)**								
Soleus	0.038 ± 0.002	0.037 ± 0.002	0.034 ± 0.001	0.031 ± 0.001	0.033 ± 0.001	0.033 ± 0.001	D**	–
Gastrocnemius	0.48 ± 0.01	0.48 ± 0.01	0.42 ± 0.01	0.41 ± 0.02	0.41 ± 0.01	0.41 ± 0.01	D***	–
**Adrenal gland relative weight (mg/100 g)**	8.36 ± 0.57	8.65 ± 0.56	7.19 ± 0.433	7.45 ± 0.59	7.55 ± 0.28	7.39 ± 0.46	D*	–
**Thymus relative weight (mg/100 mg)**	28.57 ± 0.97	26.34 ± 1.81	27.16 ± 2.15	26.55 ± 2.37	28.20 ± 2.03	24.60 ± 1.66	–	–

The two last right columns indicate the significant ANOVA results for analyzed factors (*D* Diet; *E* Exercise; *DxE* Diet*Exercise interaction; *p < 0.05; **p < 0.01; ***p < 0.001). Data are mean ± SEM. Abdominal WAT was calculated as the sum of the retroperitoneal, epididymal and mesenteric depots. WAT: White Adipose Tissue.

As for the CAFR feeding, it reverted the effects of CAF on adipose tissue depots, specifically by decreasing their absolute weight, while also decreasing the gastrocnemius absolute weight.

The analysis of obesity-associated serum parameters (Table 2) showed that CAF feeding increased glucose, triacylglycerides, insulin, HOMA-IR, leptin, adiponectin, and the leptin/adiponectin ratio compared to STD feeding. Total cholesterol and HDL-cholesterol were reduced, but LDL-cholesterol was increased in the CAF group. Exercise decreased total and HDL-cholesterol in STD and CAF fed animals, and increased leptin levels specifically in CAF animals. CAFR feeding decreased total cholesterol and leptin levels compared to CAF. The detailed results of the 1st and 2nd two-way ANOVA analyses of body composition and serum parameters are shown in supplementary Tables S3 and S4.

**Table 2:** Effects of dietary interventions and exercise on serum parameters.

	STD-C	STD-E	CAF-C	CAF-E	CAFR-C	CAFR-E	1st ANOVA (STD vs. CAF)	2nd ANOVA (CAF vs. CAFR)
Glucose (mg/L)	1027 ± 21	1083 ± 38	1186 ± 37	1125 ± 44	1143 ± 42	1071 ± 29	D**	–
Triacylglycerides (mg/L)	1175 ± 127	1216 ± 201	1688 ± 183	1830 ± 208	1466 ± 198	1611 ± 208	D**	–
Cholesterol (mg/L)	872 ± 45	742 ± 45	746 ± 31	686 ± 39	661 ± 31	596 ± 39	D*, E*	D*
LDL-Cholesterol (mg/L)	193 ± 17	224 ± 28	246 ± 27	261 ± 19	244 ± 39	218 ± 28	D*	–
HDL-cholesterol (mg/L)	785 ± 41	672 ± 25	601 ± 31	522 ± 35	540 ± 22	492 ± 26	D***, E*	–
NEFAs (mM)	0.72 ± 0.05	0.64 ± 0.04	0.65 ± 0.06	0.62 ± 0.03	0.62 ± 0.02	0.63 ± 0.04	–	–
Insulin (µg/L)	1.93 ± 0.20	1.56 ± 0.29	3.36 ± 0.35	3.76 ± 0.50	2.95 ± 0.46	2.89 ± 0.36	D***	–
HOMA-IR	12.27 ± 1.30	17.72 ± 7.74	24.54 ± 2.90	25.18 ± 2.80	21.05 ± 3.64	19.23 ± 2.65	D***	–
Leptin (ng/mL)	31.47 ± 2.84	30.01 ± 8	59.42 ± 5.33	78.38 ± 6.36	59.54 ± 4.14	54.91 ± 6.65	D***, DxE**	D*, DxE*
Adiponectin (µg/mL)	25.75 ± 1.37	23.29 ± 1.07	28.68 ± 1.32	30.83 ± 1.59	29.67 ± 1.13	28.22 ± 1.86	D**	–
Leptin/adiponectin ratio	1.25 ± 0.12	1.28 ± 0.34	2.07 ± 0.17	2.53 ± 0.15	2.04 ± 0.17	2.03 ± 0.32	D***, DxE**	–

The two rightmost columns indicate the significant ANOVA results for analyzed factors (*D* Diet; *E* Exercise; *DxE* Diet*Exercise interaction; *p < 0.05; **p < 0.01; ***p < 0.001). Data are mean ± SEM. NEFAs Non-sterified fatty acids; HOMA-IR Homeostatic model assessment-estimated insulin resistance.

CAFR diet differed significantly from STD diet in all the variables showing CAF versus CAFR differences in the 2nd two-way ANOVA. This indicates that CAFR feeding did not recover the obesity alterations on those biometric, tissues weight and serum parameters to the level of STD feeding (see supplementary Table S6).

### CAF and CAFR feeding decreased, and exercise marginally increased circadian locomotor activity

**Figure 3:**
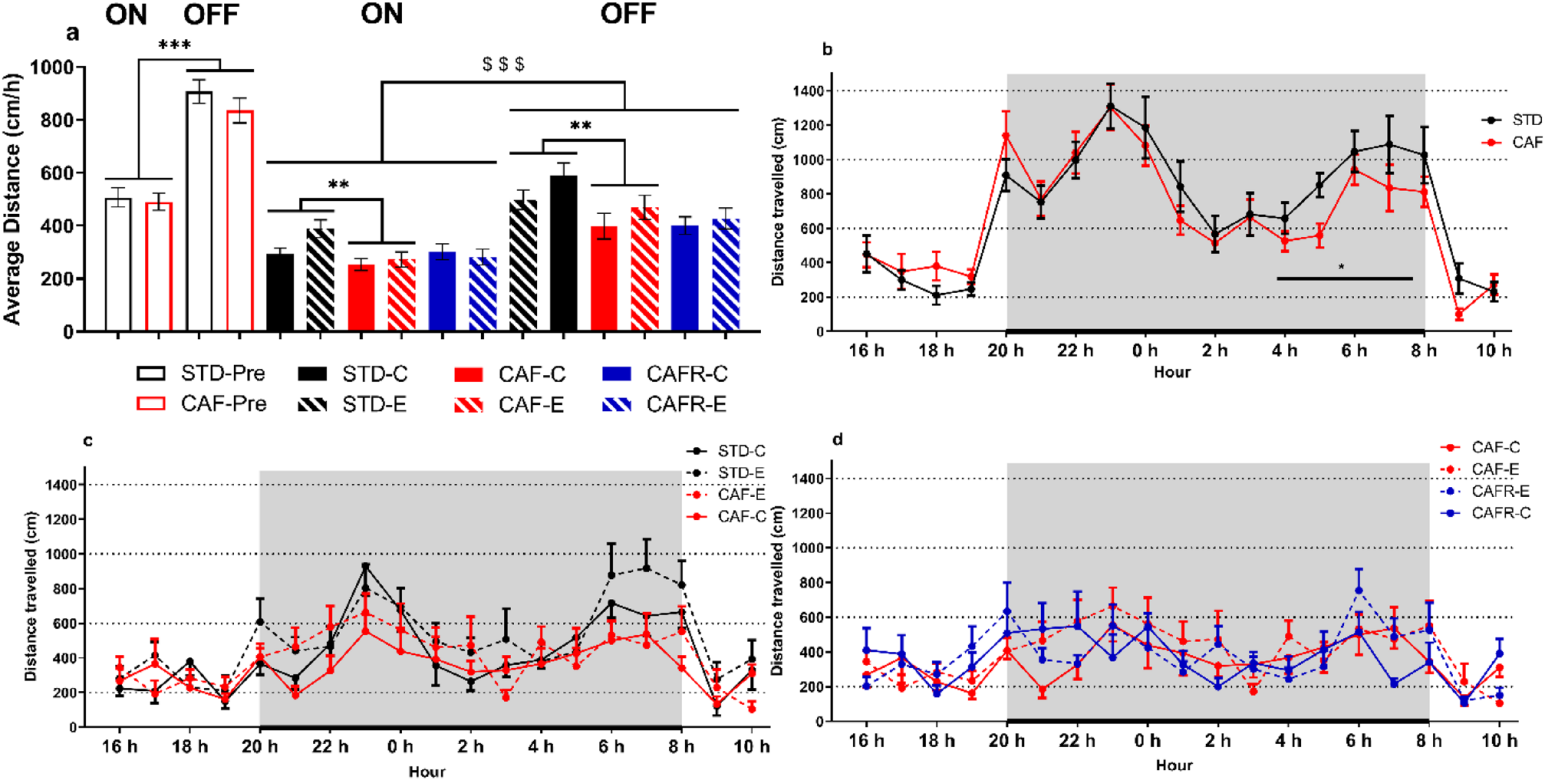
Effects of dietary interventions and exercise on circadian activity. Two Home-Cage Activity tests were performed at week 9 and week 17. (**a**) Average hourly distance traveled (cm) for the ON and OFF periods in both time points (week 9, -Pre, and week 17). (**b**) Hourly distance traveled (cm) for the ON and OFF (grey square area) periods at week 9. (**c**) Hourly distance traveled (cm) for the STD and CAF, control and exercised, groups at week 17. (**d**) Hourly distance traveled (cm) for the CAF and CAFR, control and exercised, groups at week 17. Values are mean ± SEM. $$$*p* < 0.001 versus ON period, *p < 0.05 versus STD. **p < 0.01 versus STD.

To determine whether obesity affected circadian locomotor activity, we performed the HCA test at weeks 9–10. We first analysed the average distance travelled over the lights ON and lights OFF periods (Fig. 3A). As expected, average activity in the OFF period was higher than in the ON period [period: F(1,54) = 187.851, *p* < 0.001], with no interaction with the diet. We then analysed the hourly activity over the OFF and ON periods separately. The activity changed over the OFF period hours (Fig. 3B) [hour: F(11,594) = 8.750, *p* < 0.001], but no interaction with the diet was found. However, we noticed that the CAF fed animals appeared to decrease their activity during the last 4 h of the OFF period (from 04h until 08h). So, we analysed the total activity of this 4 h-period (Fig. 3B) and found that the CAF animals were less active compared to the STD animals [U = 219; *p* = 0.016]. Regarding the ON period (Fig. 3B) we found a tendency for activity to change over time [hour: F(5270) = 2.335, *p* = 0.051], with no effect of diet.

To analyse whether the CAFR diet and/or treadmill exercise affected locomotor activity, we performed a second HCA test at week 17. We first compared the average distance travelled at weeks 9 and 17 and found an overall decrease at week 17 [F(1,50) = 148.06, *p* < 0.001]. Thus, as it can be seen in Fig. 3A, animals overall displayed an average distance reduction from 496 at week 9–10 to 300 cm/h at week 17 during the ON period, and from 860 at week 9–10 to 466 cm/h at week 17 during the OFF period.

Secondly, the average distance travelled during the ON and OFF periods were analysed (Fig. 3A). In the CAF and STD animals we found activity to increase in the OFF period compared to the ON period [period: F(1,34) = 71.314, *p* < 0.001], with no effect of diet or exercise. The between-subject factor revealed that CAF diet decreased the average distance travelled, and exercise increased it [Diet: F(1,34) = 9.564, *p* = 0.004; Exercise: F(1,34) = 5.241, *p* = 0.028; and Diet*Exercise: F(1,34) = 0.634, *p* = 0.431]. The same analysis comparing CAF and CAFR animals revealed an analogous increase in activity during the OFF period [F(1,32) = 47.312, *p* < 0.001] and no effect of diet or exercise was found.

Finally, the hourly activity over the OFF and ON periods were studied separately. The analysis in CAF and STD animals (Fig. 3C) revealed activity changes over the hours of the OFF period [hour: F(11,374) = 6.287, *p* < 0.001] but not over the ON period [hour: F(5170) = 1.600, *p* = 0.165], with no effect of diet or exercise in either period. However, the between-subject factors showed that CAF feeding had decreased the level of activity in the OFF period compared to the STD group, with exercise reaching no significant effect [Diet: F(1,34) = 5.902, *p* = 0.021; Exercise: F(1,34) = 3.155, *p* = 0.085; and Diet*Exercise: F(1,34) = 0.046, *p* = 0.831]. The analysis in CAFR and CAF animals (Fig. 3D) showed activity changes over both periods [OFF hours: F(11,352) = 2.746, *p* = 0.002; ON hours: F(5160) = 2.833, *p* = 0.025], with no effect of diet. Moreover, this activity change over the ON period was significantly affected by the exercise intervention [hour*exercise interaction F(5160) = 2.917, *p* = 0.022, Fig. 3D], with exercised animals being less active at 10AM compared to non-exercised animals [t(34) = 3.830, *p* = 0.001]. No such effect of exercise was detected in the OFF period.

### CAF and CAFR feeding decreased basal corticosterone levels

We were also interested in studying whether CAF diet-induced obesity and the dietary and exercise interventions affected baseline HPA axis activity through plasmatic ACTH and corticosterone levels, both in the morning (AM) and evening (PM).

The repeated measures comparison of corticosterone and ACTH levels between the PM and AM samples in CAF and STD animals showed an increase for both hormones in the PM [corticosterone: F(1,35) = 124.607, *p* < 0.001; ACTH: F(1,33) = 114.426, *p* < 0.001] with no effect of diet or exercise, but the between-subject effect indicated CAF animals had lower corticosterone levels than STD [Diet: F(1,35) = 9.247, *p* = 0.004], with no effect of exercise. No such effect was observed for ACTH. When we analysed the effects of CAFR feeding compared to CAF, the results showed that the CAFR group maintained the decrease in corticosterone levels.

**Figure 4:**
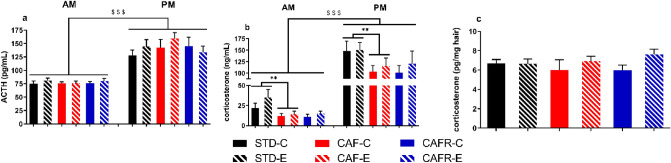
Effects of dietary interventions and exercise on circadian baseline levels of plasmatic corticosterone and ACTH, and hair corticosterone levels. (**a**) ACTH (pg/mL). (**b**) Corticosterone (ng/mL). (**c**) Hair corticosterone (pg/mg hair). Values are mean ± SEM. $$$*p* < 0.001 versus AM levels; **p < 0.01 versus STD.

We also aimed to determine long-term effects of the interventions on HPA axis activity by means of hair corticosterone levels, which have been validated as a good biomarker for evaluating the chronic activity of the HPA axis^45^. Results did not show any difference due to CAF feeding when compared to STD or any effects of exercise (Fig. 4C). On the other hand, the analysis of CAFR and CAF animals indicated no significant effects of the exercise factor [F(1,35) = 3.141, *p* = 0.086 (Fig. 4C)].

### HPA-axis response to a novel environment was unaffected by diet or exercise

**Figure 5:**
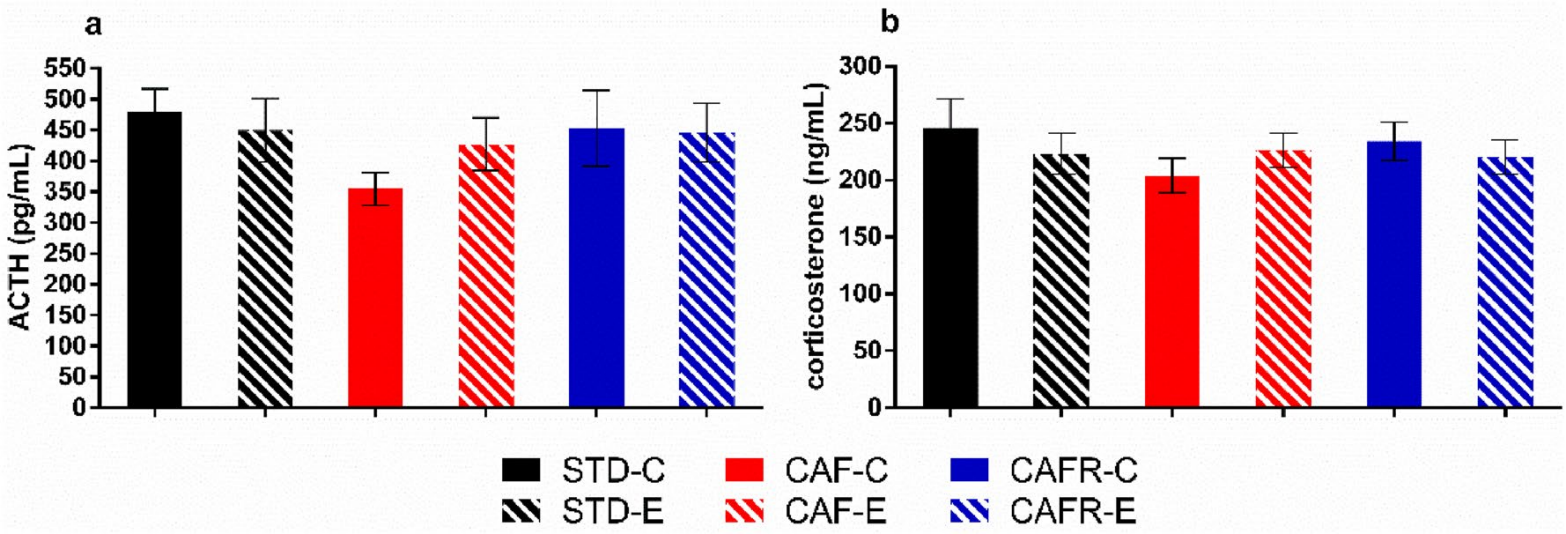
Effect of dietary interventions and exercise on HPA-axis hormone response to the Hole Board test. (**a**) Plasmatic ACTH levels (ng/mL) and (**b**) corticosterone levels (ng/mL) in response to exposure to a novel environment.

We also analysed the HPA axis reactivity to the exposure to a mild stressor, a novel environment. Immediately after the HB test, plasmatic ACTH and corticosterone levels increased 5,eightfold and 28,fourfold, respectively (Fig. 5A and B) compared with baseline AM hormone levels (shown in Fig. 4A and B). No significant effects appeared when analysing CAF and STD diets on ACTH [diet: F(1,38) = 3.326, *p* = 0.077] and corticosterone levels. Exercise had no significant effects on neither hormone.

CAFR feeding did not have any significant effect compared to CAF feeding.

### Exploratory activity in a novel environment and anxiety-like behavior

The analysis of the HB was performed considering both the first 5 min and the whole 15 min of the test.

**Figure 6:**
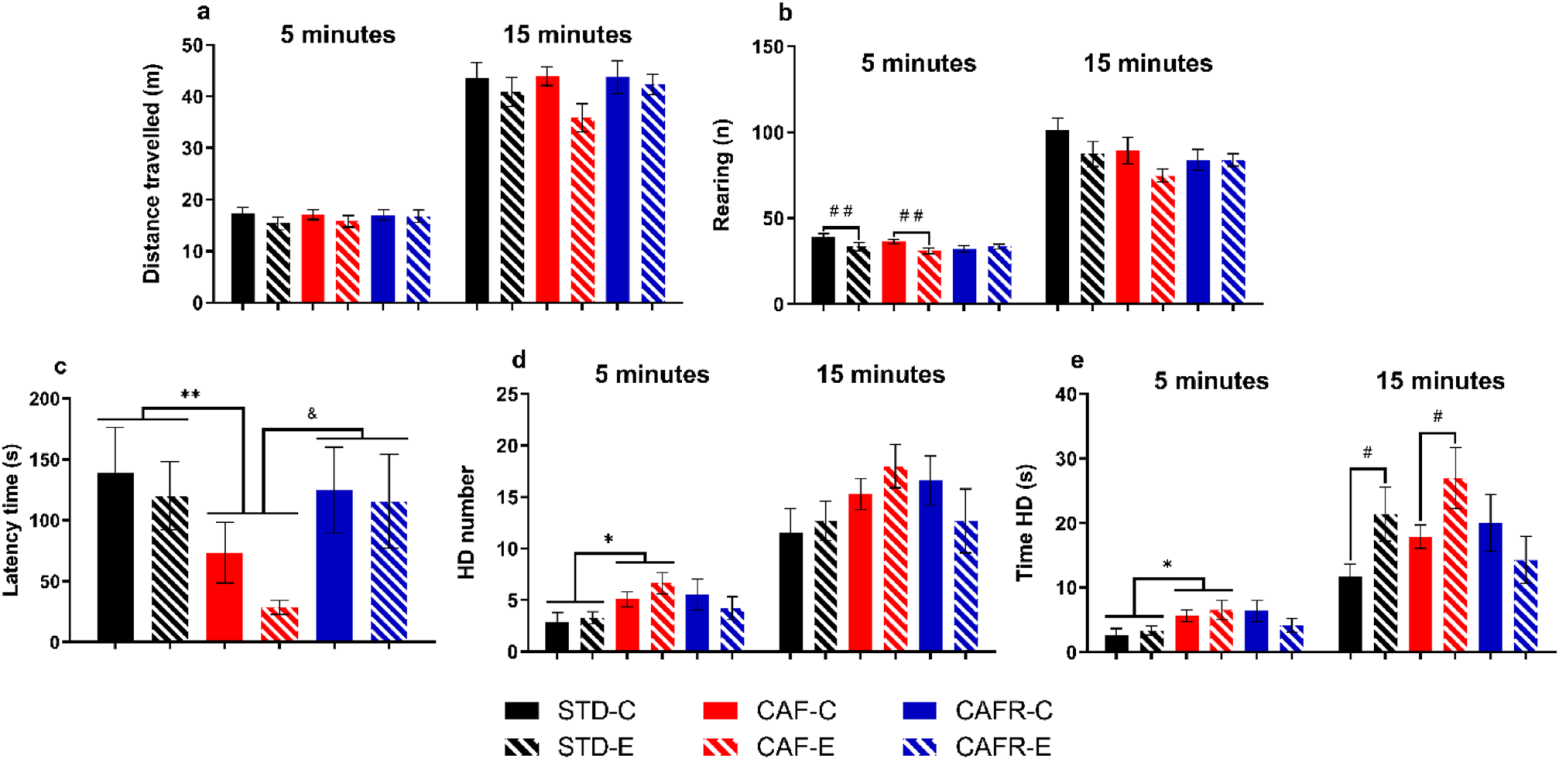
Effects of dietary treatments and exercise on ambulatory and exploratory behavior in the Hole Board test in the first 5 min and the whole 15 min test. (**a**) Distance traveled (m). (**b**) Number of rearings. (**c**) Latency time (seconds) of the first head dip. (**d**) Number of head dips. (**e**) Time spent head-dipping (seconds). Values are mean ± SEM. *p < 0.05, **p < 0.01 versus STD; & *p* < 0.05 versus CAF; #*p* < 0.05, ##*p* < 0.01 versus the respective non-exercised controls.

The analysis of the first 5 min did not reveal any effect of CAF on the distance travelled (Fig. 6A) or the rearings (Fig. 6B) compared with STD. In contrast, exercise decreased rearings [F(1,39) = 9.257, *p* = 0.004] but not distance travelled. Regarding exploratory activity, CAF decreased the latency of the first HD (Fig. 6C), and increased the number (Fig. 6D) and the time spent HD (Fig. 6E) compared with STD [latency: F(1,39) = 12.667, *p* = 0.001; number HD: F(1,39) = 11.678, *p* = 0.002; time HD: F(1,39) = 8.940, *p* = 0.005]. No effects of exercise upon these variables were detected. When we analyzed the effects of CAFR feeding compared to CAF, a significant ‘diet*exercise’ interaction on rearings was found [F(1,37) = 4.693, *p* = 0.038]. Decomposition of this interaction revealed that CAF animals performed less rearings when exercised [t(17) = 2.559, *p* = 0.020], whereas CAFR animals did not differ between exercise conditions. For exploratory activity, CAFR increased the latency of the first HD compared to CAF [F(1,37) = 5.232, *p* = 0.029] without additional significant effects/interactions in the first 5 min of the test.

For the 15-min exposure analysis, 8 animals (1 from the STD-C, CAF-C and CAFR-E groups, 2 from the CAF-E group, 3 from the STD-E group and none from the CAFR-C group) were eliminated due to them climbing the wall and escaping the apparatus. Results showed that CAF did not affect the distance travelled and the number of rearings. Exercise residually decreased the distance travelled (Fig. 6A) and the number of rearings (Fig. 6B) [distance: F(1,32) = 3.831, *p* = 0.060; rearings: F(1,32) = 4.144, *p* = 0.051]. CAF also increased the number of HD (Fig. 6D) but not the time spent HD (Fig. 6E) [number HD: F(1,32) = 4.979, *p* = 0.034; time HD: F(1,32) = 3.354, *p* = 0.078]. Exercise increased the time spent HD [F(1,32) = 8.719, *p* = 0.006], but not the number of HD. We then calculated the mean duration of a HD (time HD/number HD), and found no effect of CAF while exercise increased mean HD duration [Exercise: F(1,32) = 9.774, *p* = 0.004, data not shown]. The analysis of CAFR feeding compared to CAF revealed no diet effects on the distance travelled (Fig. 6A), the number of rearings (Fig. 6B), the number of HD (Fig. 6D) or the time spent HD (Fig. 6E). Exercise did not significantly affect the distance travelled [F(1,33) = 3.422, *p* = 0.075], nor the number of rearings [F(1,33) = 1.518, *p* = 0.228].

Finally, the EPM analysis (Fig S5) revealed no major effect of diet in anxiety-like behavior, and an effect where exercise decreased enclosed arm entries in CAF and CAFR fed animals [F(1,36) = 4.551, *p* = 0.041].

## Discussion

In this work we have shown that CAF feeding successfully induced obesity, changed body composition by increasing adiposity, and impaired metabolic health by increasing MetS blood biomarkers and adipokine levels. This dietary pattern also resulted in a sedentary profile assessed by the HCA test, reduced baseline HPA axis activity through decreased corticosterone levels, and boosted exploratory behavior but did not affect anxiety-like behavior. The decreased energy intake of CAFR animals ameliorated some biometric and metabolic parameters, but it was not sufficient to revert neither the CAF-induced sedentary profile nor the boosted exploratory activity. Regarding exercise effects, results showed that it decreased adiposity and cholesterol levels, increased baseline locomotor activity in the HCA test, and further boosted exploratory behavior while partially decreasing anxiety-like behavior.

Additionally, CAF feeding altered circadian locomotor activity. The activity pattern of control STD animals during the night hours was characterized by an increase of baseline activity in the first and last night hours and an intervening period of decreased activity, as reported previously^46^. After 10 weeks of CAF feeding, we observed a relatively mild decrease in activity during a subset of hours at the end of the night phase. However, after 17 weeks of CAF feeding the decrease of HCA was noticeable over the whole night phase. No changes in activity were observed during the light hours. In line with these results, high-fat (HF) feeding in rats has been reported to decrease activity during night hours and increase activity during day hours^47^. Baseline locomotor activity is known to be directly under control of the master clock in the suprachiasmatic nucleus^48^ and it has been reported that knock-out of the Clock gene in mice increased daytime feeding along with alterations in activity^49^. Alterations in circadian rhythm have been shown to occur in HF diet mice, including attenuation of diurnal rhythms of feeding and locomotor activity, and attenuation of the expression of circadian clock genes and diurnal patterns of metabolic markers^4^. In our case, CAF feeding increased energy intake, but whether this altered eating pattern was associated with increased daytime feeding should be further explored in future studies.

Besides analysing the locomotor activity pattern, we also wanted to determine whether CAF feeding affected the circadian cycle of the HPA axis. Plasmatic ACTH and corticosterone levels showed a circadian cycle with higher hormone levels in the evening and lower levels in the morning, as expected^50^. CAF feeding did not alter this pattern but decreased basal corticosterone levels both in the morning and evening. This result is consistent with another study in which decreased corticosterone and no effect on ACTH baseline levels were obtained after 5 weeks of CAF feeding in rats^51^. The fact that there were no differences in baseline circulating ACTH could indicate that the CAF-induced effects could be exerted at the level of the adrenal gland. In mice, increased corticosterone levels, cortical adrenal hyperplasia and increased Melanocortin-2 Receptor expression have been reported after long-term HF feeding^52^. Although we found a decrease in relative adrenal gland weight in CAF fed animals which might partially explain the decreased corticosterone basal levels, absolute weight was unaffected, and we cannot discard changes in ACTH signalling being the cause of the decrease in baseline corticosterone. In humans there is no consensus about baseline levels of cortisol in obesity^53^, with reports showing increased^54^ or decreased^55^ levels, and similar results can be found for ACTH levels^56,57^. Additionally, we determined corticosterone concentration in hair, which is used as a biomarker of long-term HPA axis activity and thought to reflect free corticosterone^45^, which is considered as the bioavailable portion^58^. The fact that we did not find differences in hair corticosterone between STD and CAF-fed animals suggests that CAF feeding might not alter bioavailable corticosterone levels. This might indicate that although total corticosterone levels would be decreased due to the CAF diet, compensatory mechanisms might be at play in generating adequate levels of free corticosterone and therefore a normal adrenal function in the obese state. It is important to note that RIA measures are an indication of total corticosterone levels thus reflecting axis activity rather that bioavailable free circulating corticosterone.

We also determined the effects of a mild stress challenge, novel environment exposure, on the HPA axis response. We detected a slight decrease in ACTH levels in CAF-fed animals. This is in accordance with other studies showing that palatable food intake, even in short-term exposures and without body weight gain, decreased ACTH response to restraint stress and anxiety-like behavior in the EPM^18^, which was attributed to a stress-buffering action of the hedonic properties of palatable foods mediated by reward-induced structural plasticity in the basolateral amygdala. The small effect of CAF feeding observed here might be related with the type of stimulus, since restraint stress or forced swim generate a much greater response than novel environment exposure^59^. On the other hand, we have not found differences in corticosterone levels in response to a mild stressor suggesting that the HPA axis can respond normally to this kind of challenge by regulating small changes in hormone levels.

At the behavioral level, CAF feeding induced an increase in exploratory activity, in accordance with a previous study from our group^9^. Interestingly, other rodent models of DIO in which animals were fed a non-palatable HFD reported decreased exploratory activity^60,61^, which suggests differential effects of obesity-induced diets depending on their palatability. Additionally, CAF feeding did not alter anxiety-like behavior in the EPM. In a previous study we have shown that 8 weeks of post-weaning CAF feeding had no effects on anxiety-like behavior^9^, and this result seems to be replicated after 18 weeks.

After characterizing the effects that CAF feeding had compared to STD, we also studied the effects that the restricted CAF diet had on obese animals. The decrease in energy intake observed in CAFR compared to CAF was reflected in the AC, which was diminished in CAFR animals. CAFR feeding also decreased the weight of subcutaneous and abdominal adipose tissue compared to CAF. This decreased adiposity, which was not accompanied by a reduced body weight, was reflected in lower leptin levels in serum. Moreover, CAFR feeding also changed body composition by slightly decreasing the gastrocnemius muscle absolute weight. Interestingly, CAFR feeding decreased mainly the abdominal tissue depots, but not the subcutaneous, which suggests an adipose tissue redistribution that has been associated with decreased cardiovascular risk in humans^62^. Other markers of MetS, such as serum total cholesterol levels, were also decreased in CAFR animals. Therefore, CAFR feeding intervention would be associated with better health outcomes. However, this diet did not significantly revert glucose dysregulation induced by the CAF diet, showing similar glucose and insulin levels. It could be hypothesized that a longer CAFR intervention would revert the effects of CAF feeding on glucose dysregulations. It is also important to note that in this study the interventions were of a corrective nature, which are considered less effective than preventive interventions^63^. In fact, CAFR feeding administered post-weaning showed a greater amelioration of glucose and insulin levels, and of HOMA-IR compared to CAF^27^ than in the present study.

Despite the amelioration of the biometric parameters and adiposity, CAFR diet did not change the HCA compared to CAF, indicating that the dysregulation of the circadian locomotor activity pattern induced by CAF feeding, consisting in a more sedentary pattern, is not corrected by the CAFR diet. As for the baseline corticosterone and ACTH levels, we did not find differences between CAF and CAFR feeding. Mouse studies in which a HFD was interrupted after 6 weeks reported increased baseline corticosterone levels, indicating a withdrawal response to the preferred diet^64^. We did not observe such an effect, which suggests that the decrease of energy intake resulting from the CAF to CAFR feeding shift did not generate this withdrawal-like effect. We have previously shown that CAFR feeding induced changes in feeding behavior by increasing the proportion of regular chow that the animals consume, compared to the amount of chow consumed while CAF feeding, which indicates that those animals ate chow *a.l.* and were not starved^27,28^. Hair corticosterone levels were also not different in CAFR fed animals when compared to CAF fed animals.

HPA response to a novel environment was also not affected by CAFR feeding, indicating that the effects of CAF feeding are also present in CAFR feeding. Behaviorally, CAFR fed animals also displayed similar levels of exploratory and anxiety-like behaviors to CAF fed animals. In another study, mice fed a palatable diet and switched to standard chow after 6 weeks decreased the time spent in the open arms of the EPM, thus reflecting increased anxiety-like behavior^64^. Moreover, in rats switched to chow after 15 weeks of cafeteria diet, gene expression of CRH increased suggesting increased stress^65^. We have shown the CAFR diet is capable of increasing chow intake when administered post-weaning^27,28^. However, in our study we did not detect the increased stress and anxiety measures that are associated with diet withdrawal.

In this study we aimed to evaluate the effects of an exercise intervention. Regarding its effects in CAF-fed animals compared to STD animals, exercised animals presented decreased adiposity in the retroperitoneal fat depot and remarkably, decreased total abdominal adiposity, which points to a healthier body composition profile since, as mentioned above, abdominal adiposity rather than subcutaneous adiposity is associated with MetS^66^. This result is interesting because the treadmill protocol used in the present study, consisting of 12 m/min, 35 min/session and 5 sessions/week, is considered of moderate intensity, equivalent to brisk walking or slow running in humans^67^. However, this exercise did not affect the body weight, Lee Index or AC. Although similar treadmill interventions (12 m/min for 45 min) have resulted in ~ 25% decreased in CAF induced body weight gain^68^, greater exercise intensities, such as 20 m/min for 1 h at 5% incline, or different paradigms, such as voluntary wheel running, seem to be necessary to consistently ameliorate biometric parameters^69,70^. Regardless, exercise improved body composition and reduced abdominal adiposity, without affecting energy intake. This is consistent with other studies using a similar exercise protocol in CAF fed animals^68^. Even when a higher exercise intensity (15 m/min for 1 h) was applied to chow-fed rats, no effect was reported on energy intake, although several changes of eating patterns such as decreased meal size and increased meal frequency during the light phase have been described^71^. Exercise was able to decrease cholesterolemia, indicating an amelioration of metabolic risk biomarkers. Exercise also diminished absolute weights (inguinal and total) and relative weights (inguinal, retroperitoneal and total) of adipose depots, and total serum cholesterol levels in animals fed with standard chow or the CAFR diet. This is in line with previous results in which treadmill exercise showed beneficial effects both in obese and non-obese animals^36,38^. The new finding in the present report is that these effects are also present in animals fed a restricted-cafeteria diet.

As for the effects of treadmill exercise on HCA, we found an increase during the night hours both in the STD- and CAF-fed groups. This result is consistent with another study reporting that voluntary wheel running in rats resulted in weight loss, reduced daytime feeding, and reverted the effects of a HFD on circadian locomotor activity^70^. However, it is unclear whether this improvement in circadian regulation was due to the exercise itself or to the weight loss. The fact that we observed a partial reversion of the sedentary profile induced by CAF feeding without reducing body weight suggests that exercise by itself would be capable of improving circadian function in DIO rats, although it did not fully restore the activity pattern seen in STD-fed animals. This might indicate that while treadmill exercise can partially increase activity, its effects are milder that voluntary wheel running^70^. We have not found any reports of similar treadmill exercise protocols in which baseline circadian locomotor activity was measured.

Exercise did not affect the baseline HPA axis activity. In a previous study using long-term moderate treadmill exercise exposures (12 m/min for 30 min) we have also reported no effect on baseline corticosterone^38^. On the other hand, treadmill exercise at a higher intensity than ours (15 m/min for 60 min) has shown increased corticosterone levels after 2 weeks of training^39^. In other exercise paradigms such as wheel running, similar results have been found, with baseline corticosterone levels increasing after a short-term exposure to the exercise that were normalized after 4 weeks^72^. Taken together this suggests that baseline activity of the HPA axis may increase because of exercise in the short term but reverts to control levels in the long term. This response might be similar to that found in paradigms of chronic predictable stress. Thus, daily immobilization stress for 7 days generated increased baseline corticosterone levels^73^, while a similar but milder paradigm lasting 4 weeks did not^74^.

Even though we detected no effects of exercise upon plasmatic levels of corticosterone, exercise tended to increase hair corticosterone in CAF and CAFR. This might indicate that for obese animals, treadmill exercise could be a more challenging stimulus requiring more effort compared to lean STD animals. Single stressful situations that might increase corticosterone acutely are masked in integrated measures such as hair corticosterone, but repeated stressful situations generate an increase in hair corticosterone ^45^. There are reports of DIO rats having lower exercise endurance and failing to adequately perform treadmill exercise earlier than control animals at high intensities (25 m/min)^75^, which might indicate treadmill exercise represents a greater challenge to obese animals.

Regarding the effects of exercise on the HPA axis response to a mild novel environment stress challenge, in a previous study mentioned above we have shown that long-term treadmill exercise initiated post-weaning (12 m/min for 30 min/day for 33 weeks) can decrease the ACTH response to a novel environment exposure in Sprague Dawley rats^38^. In the present study this effect was not replicated, which might be related with the duration of the treadmill intervention (33 weeks vs 8 weeks). It might also be related to the stage of development of the animals in which the exercise was performed, since the exercise in the present study started in a later stage when animals were already adults. Another possible factor lies in the rat strain used. Here we have used Long-Evans rats, which are known to present higher resting levels of ACTH and corticosterone, as well as a higher hormonal response to the HB^76^ compared to Sprague–Dawley rats.

Treadmill exercise increased exploratory activity in STD-fed animals and further enhanced the already increased exploratory activity induced by CAF feeding. Additionally, exercise marginally decreased anxiety-like behavior, which is consistent with previous reports from our group^9^. There are reports of exercise increasing anxiety behavior in chow-fed rats in a different behavioural test^71^, or of no effect of treadmill exercise^77^. Several variables such as the use of electric stimulus to motivate running as well as exercise intensity, duration, and paradigm (treadmill vs wheel running) might generate different behavioral profiles ^36,77^. In our exercise protocol control animals were placed in the treadmill daily as a control for handling and exposure to the apparatus, this implies that both exercised and control groups were handled daily which can induce an anxiolytic effect^78^ and mask the effect of exercise, as we have also shown previously^38^.

To conclude, restricted CAF feeding reverted, in part, the effects of CAF-induced obesity on biometric parameters, adiposity, and metabolic biomarkers, but it was not able to revert the sedentary profile and the decrease in baseline corticosterone levels. Exercise also decreased adiposity, partially corrected metabolic alterations and increased baseline locomotor activity and exploratory behavior in STD and CAF animals, but not in CAFR. Therefore, exercise appears to have beneficial effects in reverting the alterations of CAF, but it does not seem to show additional benefits when paired with CAFR.

## Supplementary Information

Supplementary Information.

## Data Availability

The datasets generated during and/or analyzed during the current study are available from the corresponding author on reasonable request.
