# Supplementary material for: Restricted cafeteria feeding and treadmill exercise improved body composition, metabolic profile and exploratory behavior in obese male rats
Source: Sci Rep. 2022 Nov 15;12:19545. doi: 10.1038/s41598-022-23464-7 (PMC9666649; doi:10.1038/s41598-022-23464-7)
Supplement: Supplementary file 1 — Supplementary Information. [file 41598_2022_23464_MOESM1_ESM.pdf]

Effects of restrictive cafeteria feeding and treadmill exercise on body composition, metabolic profile, locomotor activity, exploratory behaviour and HPA axis in obese rats.

Adam Alvarez-Monell[1,4], Alex Subias-Gusils[2,4], Roger Mariné-Casadó[6], Xavier Belda[3,4], Humberto Gagliano[3,4], Oscar J Pozo[5], Noemí Boqué[6], Antoni Caimari[6], Antonio Armario[3,4], Montserrat Solanas[1,4,*], and Rosa M Escorihuela[3,4,*]

[1]Medical Physiology Unit, Department of Cell Biology, Physiology and Immunology, Faculty of Medicine, Universitat Autònoma de Barcelona, 08193 Bellaterra, Spain.

[2]Unitat de Psicologia Mèdica, Departament de Psiquiatria i Medicina legal, Universitat Autònoma de Barcelona, 08193 Bellaterra, Spain.

[3]Animal Physiology Unit, Department of Cell Biology, Physiology and Immunology, Faculty of Biosciences, Universitat Autònoma de Barcelona, 08193 Bellaterra, Spain.

[4]Institut de Neurociències, Universitat Autònoma de Barcelona, 08193 Bellaterra, Spain.

[5]Gastroesophageal Carcinogenesis Group, IMIM (Hospital del Mar Medical Research Institute), Carrer Doctor Aiguader 88, 08003, Barcelona, Spain.

[6] Eurecat, Centre tecnològic de Catalunya, Biotechnology Area and Technological Unit of Nutrition and Health, 43204 Reus, Spain

*Montserrat.Solanas@uab.cat

*Rosamaria.Escorihuela@uab.cat

Supplementary Table S1. Cafeteria (CAF) diet composition for the first period of the experiment.

Ingredient	Week 1	Week 4	Week 8
**Muffin**	3,25 g	7 g	7 g
**Bacon**	3 g	6 g	8 g
**Carrot**	2,5 g	6 g	8 g
**Biscuits with pâté**	3,5 g	5 g	5 g
**Biscuits with cheese**	3,5 g	5 g	5 g
**Jellied sugared milk**	25 g	40 g	45 g
**Chow**	15 g	15 g	25 g
**g of diet provided**	56 g	84 g	103 g
**Kcal provided**	131	197	246

Supplementary Table S2. Detailed treadmill training protocol for the progressive intensity increase from session 1 (week 1) until session 12 (week 2)

Minute	S.1	S.2	S.3	S.4	S.5 – S.7	S.8 – S.12	S.13 onwards
**0**	0 m/min	0 m/min	4 m/min	4 m/min	5 m/min	5 m/min	6 m/min
**1**	0 m/min	0 m/min	5 m/min	5 m/min	6 m/min	6 m/min	7 m/min
**2**	0 m/min	0 m/min	6 m/min	6 m/min	7 m/min	7 m/min	8 m/min
**3**	0 m/min	0 m/min	7 m/min	7 m/min	8 m/min	8 m/min	9 m/min
**4**	0 m/min	0 m/min	8 m/min	8 m/min	9 m/min	9 m/min	10 m/min
**5**	0 m/min	4 m/min	9 m/min	9 m/min	10 m/min	10 m/min	11 m/min
**6**	0 m/min	5 m/min	9 m/min	9 m/min	10 m/min	11 m/min	12 m/min
**7**	0 m/min	6 m/min	9 m/min	9 m/min	10 m/min	11 m/min	12 m/min
**8**	0 m/min	7 m/min	9 m/min	9 m/min	10 m/min	11 m/min	12 m/min
**9 to 14**	0 m/min	8 m/min	9 m/min	9 m/min	10 m/min	11 m/min	12 m/min
**15**	4 m/min	8 m/min	9 m/min	10 m/min	10 m/min	11 m/min	12 m/min
**16**	5 m/min	8 m/min	9 m/min	10 m/min	10 m/min	11 m/min	12 m/min
**17**	6 m/min	8 m/min	9 m/min	10 m/min	10 m/min	11 m/min	12 m/min
**18**	7 m/min	8 m/min	9 m/min	10 m/min	10 m/min	11 m/min	12 m/min
**19 to 24**	8 m/min	8 m/min	9 m/min	10 m/min	10 m/min	11 m/min	12 m/min
**25 to 29**	8 m/min	8 m/min	9 m/min	9 m/min	10 m/min	11 m/min	12 m/min
**30 ot 35**	8 m/min	8 m/min	7 m/min	8 m/min	8 m/min	8 m/min	8 m/min
**Total distance (feet)**	400 ft	660 ft	860 ft	920 ft	1000 ft	1100 ft	1250 ft
**Total distance (meters)**	122 m	201 m	262 m	280 m	305 m	335 m	381 m

Supplementary Table S3. Statistical results for the ANOVA analysis of tissue weight

	STD-CAF						CAF-CAFR					
	DIET		EXE		DIET*EXE		DIET		EXE		DIET*EXE	
	F (1,34)	p	F(1,34)	p	F (1,34)	p	F(1,32)	p	F(1,32)	p	F(1,32)	p
Inguinal WAT (g)	65,156	<0,001	0,000	0,994	1,030	0,317	4,711	0,038	0,814	0,374	3,464	0,072
Retroperitoneal WAT (g)	141,496	<0,001	3,527	0,069	0,252	0,619	9,314	0,005	1,099	0,302	0,023	0,881
Mesenteric WAT (g)	51,068	<0,001	2,133	0,153	0,000	0,996	6,710	0,014	0,649	0,427	0,721	0,402
Epididymal WAT (g)	135,051	<0,001	0,277	0,602	1,252	0,271	5,239	0,029	0,003	0,955	0,364	0,550
Visceral WAT (g)	137,092	<0,001	2,585	0,117	0,315	0,578	9,707	0,004	0,702	0,408	0,027	0,870
Total WAT (g)	102,576	<0,001	0,566	0,457	0,722	0,401	7,956	00,008	0,906	0,348	1,099	0,302
Soleus muscle (g)	0,100	0,754	1,304	0,261	0,659	0,423	0,737	0,397	1,695	0,202	0,580	0,452
Gastrocnemius muscle (g)	0,759	0,390	0,423	0,520	0,204	0,654	5,014	0,032	0,768	0,387	0,009	0,923
Adrenal gland (mg)	0,064	0.802	0,201	0,657	0,011	0,918	0,229	0,636	0,009	0,924	0,515	0,478
Thymus (mg)	3,806	0,059	0,563	0,458	0,103	0,751	0,805	0,376	1,472	0,234	0,665	0,421
Inguinal WAT (g/100g)	55,137	<0,001	0,022	0,883	1,424	0,241	2,714	0,109	1,027	0,319	4,012	0,054
Retroperitoneal WAT (g/100g)	136,984	<0,001	6,169	0,018	0,622	0,436	6,697	0,014	1,193	0,283	0,135	0,716
Mesenteric WAT (g/100g)	32,888	<0,001	3,546	0,068	0,013	0,912	5,231	0,029	0,783	0,383	2,398	0,131
Epididymal WAT (g/100g)	118,349	<0,001	1,162	0,289	3,326	0,077	1,793	0,190	0,096	0,758	0,223	0,640
Visceral WAT (g/100g)	142,560	<0,001	5,479	0,025	0,837	0,367	7,541	0,010	0,758	0,391	0,289	0,595
Total WAT (g/100g)	100,955	<0,001	1,323	0,258	1,348	0,254	5,776	0,022	1,198	0,282	1,224	0,277
Soleus muscle (g/100g)	11,586	0,002	1,251	0,271	0,823	0,371	0,011	0,916	1,519	0,227	1,514	0,228
Gastrocnemius muscle (g/100g)	31,851	<0,001	0,100	0,753	0,186	0,669	0,254	0,618	0,091	0,765	0,256	0,616
Adrenal gland (mg/100g)	4,652	0,038	0,240	0,627	0,001	0,977	0,109	0,743	0,010	0,922	0,207	0,652
thymus (mg/100g)	0,105	0,748	0,579	0,452	0,188	0,668	0,047	0,829	1,032	0,317	0,620	0,476

Supplementary Table S4. Statistical results for the ANOVA analysis of metabolic serum parameters

	STD-CAF						CAF-CAFR					
	DIET		EXE		DIET*EXE		DIET		EXE		DIET*EXE	
	F (1,32)	p	F(1,32)	p	F (1,32)	p	F(1,32)	p	F(1,32)	p	F(1,32)	p
Glucose (mg/L)	11,900	0,002	0,618	0,438	1,230	0,276	1,570	0,219	2,940	0,096	0,019	0,892
Triacylglycerides (mg/L)	13,250	0,001	0,001	0,973	0,618	0,437	1,211	0,279	0,513	0,479	0,000	0,993
Cholestherol (mg/L)	4,402	0,044	5,044	0,032	0,759	0,390	5,920	0,021	3,034	0,091	0,005	0,945
LDL-Cholesterol (mg/L)	6,242	0,018	0,240	0,628	0,035	0,852	0,625	0,435	0,035	0,852	0,485	0,491
HDL-cholesterol (mg/L)	22,162	0,000	6,141	0,019	0,062	0,805	2,455	0,127	4,710	0,038	0,287	0,596
NEFAs (mM)	0,835	0,368	0,922	0,344	0,135	0,715	0,019	0,890	0,022	0,882	0,313	0,580
insulin (µg/L)	25,705	0,000	0,003	0,959	1,155	0,291	2,225	0,146	0,163	0,689	0,289	0,594
HOMA-IR	33,689	0,000	0,094	0,762	0,336	0,566	2,292	0,140	0,036	0,851	0,157	0,695
Leptin (ng/mL)	77,574	0,000	0,856	0,362	9,803	0,004	4,195	0,049	1,578	0,218	4,280	0,047
Adiponectin (µg/mL)	13,053	0,001	0,052	0,821	3,071	0,089	0,294	0,591	0,053	0,819	1,437	0,239
Leptin/adiponectin ratio	68,517	0,000	0,164	0,688	8,048	0,008	1,551	0,222	1,134	0,295	1,165	0,288

Supplementary Figure S5. Exercise decreased activity in the enclosed arms without affecting the open arms.

The first analysis of the EPM variables to evaluate the effects of CAF feeding and exercise upon total entries (Fig. 8.A), total distance travelled (Fig. 8.B) and time spent in the centre of the maze (Fig. 8.C) revealed CAF feeding to decrease the time spent in the centre of the maze [F(1,37)=5.978, p=0.020] (Fig. 8C). No other effects/interactions on these variables were found. The second analysis for comparing these variables between CAF and CAFR diet and exercise also revealed no effects/interactions

We then proceeded to analyse the number of entries (Fig. 8.A), the distance travelled (Fig. 8.B) and the time spent into the open and enclosed arms (Fig. 8.C). The first analysis showed no effects/interactions on any of those variables. The second analysis reported an effect of exercise decreasing the number of entries and the distance travelled in the enclosed arms [entries: F(1,36)=4.551, p=0.041; distance travelled: F(1,36)=4.691, p=0.038], but not affecting the time spent in the enclosed arms. No other significant effects of the diet not 'diet*exercise' was found. These results taken together point to no major differences on anxiety-like behaviour as measured in the EPM between diet and exercise conditions

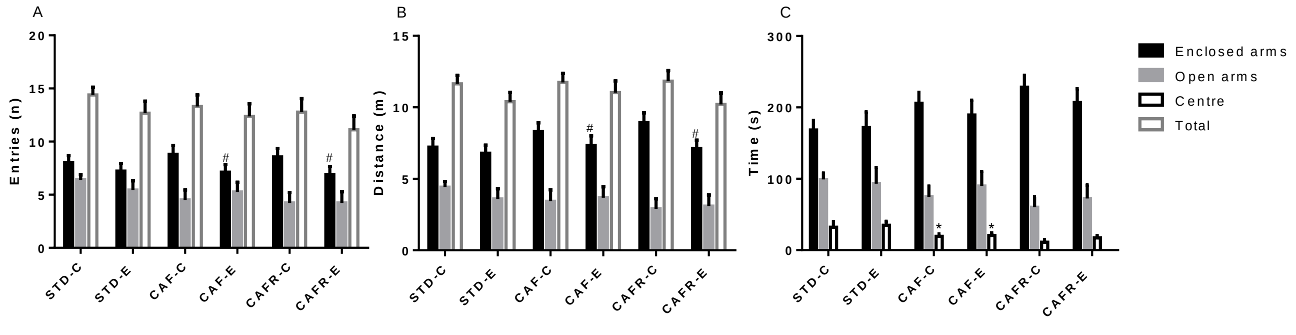

Fig. S5. Anxiety-like behaviour in the Elevated Plus Maze test. A) Number of entries. B) Distance travelled (m). C) Time spend seconds). * p<0.05 vs STD; # p<0.05 vs corresponding non-exercised group.

Supplementary Table S6. Statistical results for the ANOVA analysis comparing STD and CAFR diets

	Diet			Exercise		
	fd	F	P	fd	F	P
Biometric parameters						
AC (cm)	1,37	25	<0.001	1,37	0,000	ns
Tissues						
Inguinal WAT (g)	1,37	50,625	<0.001	1,37	5,811	**0,021**
Retroperitoneal WAT (g)	1,37	89,701	<0.001	1,37	3,696	0,063
Mesenteric WAT (g)	1,37	28,484	<0.001	1,37	0,612	ns
Epididymal WAT (g)	1,37	157,945	<0.001	1,37	2,636	ns
Abdominal WAT (g)	1,37	94,63	<0.001	1,37	2,727	ns
Total WAT (g)	1,37	84,802	<0.001	1,37	5,199	**0,029**
Gastrocnemius (g)	1,37	3,922	0,056	1,37	0,413	ns
Retroperitoneal WAT (g/100g	1,37	74,148	<0.001	1,37	4,15	**0,049**
Mesenteric WAT (g/100g)	1,37	17,273	<0.001	1,37	0,596	ns
Abdominal WAT (g/100g)	1,37	81,176	<0.001	1,37	3,122	0,086
Total WAT (g/100g)	1,37	70,085	<0.001	1,37	5,876	**0,021**
Gastrocnemius (g/100g)	1,37	59,578	<0.001	1,37	0,037	ns
Serum parameters						
Leptin (ng/mL)	1,37	20,727	<0.001	1,37	0,274	ns
Cholesterol (mg/L)	1,37	18,654	<0.001	1,37	5,515	**0,025**
